# Combination of contrast with stress echocardiography: A practical guide to methods and interpretation

**DOI:** 10.1186/1476-7120-2-15

**Published:** 2004-08-26

**Authors:** Stuart Moir, Thomas H Marwick

**Affiliations:** 1 Division of Medicine, University of Queensland, Brisbane, Australia

## Abstract

Contrast echocardiography has an established role for enhancement of the right heart Doppler signals, the detection of intra-cardiac shunts, and most recently for left ventricular cavity opacification (LVO). The use of intravenously administered micro-bubbles to traverse the myocardial microcirculation in order to outline myocardial viability and perfusion has been the source of research studies for a number of years. Despite the enthusiasm of investigators, myocardial contrast echocardiography (MCE) has not attained routine clinical use and LV opacification during stress has been less widely adopted than the data would support. The purpose of this review is to facilitate an understanding of the involved imaging technologies that have made this technique more feasible for clinical practice, and to guide its introduction into the practice of the non-expert user.

## I. Imaging principles

### Micro-bubbles

Two aspects of micro-bubbles are important – their gas content and the nature of their shell. Recently-approved micro-bubbles almost universally involve encapsulation of a high molecular weight gas, which improves the persistence of the bubble, optimising the number available in the left heart chambers. Air has a greater propensity to dissolve into solution and although currently unattractive because of loss of gas before arrival on the right side of the heart, better encapsulation may allow its resurgence – the benefit would be more rapid disappearance when the bubble bursts. The nature of the shell or surface modifying agent, which improves stability and prevents dissolution, may become important for new targeted imaging approaches.

### Interaction of micro-bubbles and ultrasound

Even when optimal microbubble delivery to the myocardium is achieved with invasive coronary [1] or aortic root [2] injections, detection of reliable myocardial opacification using standard 2D imaging is difficult. Whilst this partly reflects difficulty distinguishing bright, grey scale echo signals from the myocardial tissue from those of micro-bubbles within the myocardial micro-circulation, the problem is multi-factorial and has been overcome by the development of contrast specific imaging modalities, which exploit the unique interaction between the ultrasound field and micro bubbles[3] to maximise the received contrast backscatter and minimise myocardial tissue backscatter.

Micro-bubbles oscillate (expand and contract) in the ultrasound field. The pattern and nature of their oscillation, and thus the nature of the backscatter signal, differs, depending on the acoustic power of the transmitted ultrasound field, which is expressed on modern ultrasound machines as the mechanical index (MI). In general echocardiography, the amplitude of returning backscatter depends on the nature of the insonated structure, and is represented by brightness on the formed image. Most backscatter returns at the same frequency as the transmitted ultrasound (fundamental frequency).

**Figure 1:**
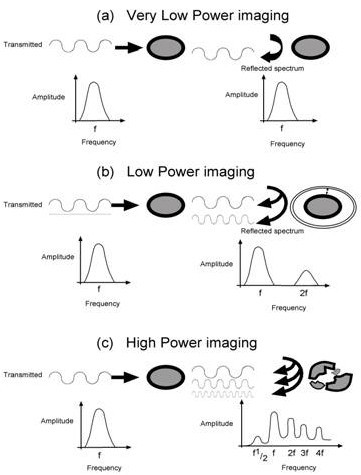
Interaction of micro-bubbles and ultrasound.

With **very low **mechanical index imaging (MI < 0.1), micro bubbles demonstrate linear oscillation, where the contraction and expansion of the micro bubble are equal. All returning micro bubble backscatter remains within the range of the frequency transmitted by the transducer (the fundamental frequency) – as for surrounding structures (Figure 1a).

**Low **mechanical index imaging (0.1 – 0.3), generates **nonlinear oscillation **of the micro bubble whereby expansion is greater than contraction. In addition to the usual backscatter of a particular amplitude within the range of the fundamental frequency, the bubbles also produce backscatter of (lesser) amplitude at **harmonic **frequencies [4,5]. For example scatter from a bubble interrogated by a 4 MHz pulse will be composed of 4 MHz and 8 MHz sound (Figure 1b). Tissues and surrounding structures do not produce harmonic backscatter when imaged under low power.

When exposed to **high **mechanical index imaging (MI > 0.6, ie the MI used for standard imaging) the bubbles oscillate wildly and burst. Upon destruction, micro-bubbles produce a brief, high amplitude signal, with good resolution, which is rich in harmonic signals, containing backscatter at the second third and fourth harmonics etc (Figure 1c). The bubble dissipates gradually following destruction, depending upon the gas composition of the bubble, with air-based micro-bubbles dissipating much more rapidly. Importantly at high MI, tissues also produce harmonics.

With standard fundamental imaging (high MI), myocardial micro-bubbles are destroyed continuously, causing apical swirling on LVO images and never replenishing the myocardium within the beam, and thus not returning significant myocardial backscatter. This exacerbates the underlying difficulty of differentiating on grey scale, between tissue backscatter and micro-bubble backscatter.

Changing to standard harmonic imaging (processing only signals returned at the second harmonic of the fundamental frequency) significantly improves LVO, but offers little benefit for myocardial perfusion, with ongoing bubble destruction and sub-optimal differentiation between contrast and tissue, because both produce harmonics at high power. Contrast specific imaging modalities, with the aim of enhancing the contrast to tissue backscatter signal ratio (CTR) are required for optimal performance of a myocardial contrast study.

### Contrast specific imaging technology

#### High Power Techniques – Intermittent Imaging

##### Triggered harmonic imaging

Porter demonstrated that intermittent high power imaging demonstrated significantly better myocardial opacification than continuous imaging, with the best opacification obtained using intermittent harmonic imaging [6]. During intermittent high power imaging, high energy ultrasound is transmitted at specified intermittent intervals, triggered to the ECG (eg once every 4 cardiac cycles; 1:4 triggering). The time between destructive pulses allows the micro-bubbles to replenish the myocardium. With each destructive pulse, high amplitude backscatter rich in harmonics is returned to the transducer, enabling static images of myocardial perfusion.

Moreover, by incrementally increasing the triggering intervals (continuous → 1:1 → 1:2 → 1:4 → 1:8 etc), the rate of replenishment of the ultrasound beam over time can be assessed both qualitatively and quantitatively.

**Figure 2:**
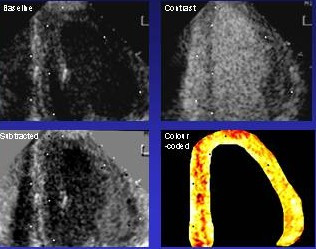
Digital subtraction and colour coding of MCE images acquired using intermittent harmonic imaging.

The qualitative assessment of perfusion using intermittent harmonic imaging can be improved by digital subtraction of the myocardial signal, and optimized by colour coding techniques to allow better extraction of bubble signals [7] (Figure 2). The first human study validating myocardial perfusion assessment by MCE with SPECT used digital subtraction [8]. However, this approach requires careful superimposition of each frame, and although the analysis is becoming more automated, the processing is still performed off-line.

##### Harmonic Power Doppler

Power Doppler technology is designed to detect motion of blood or tissue, and is used in myocardial contrast echo to overcome the need for off line digital subtraction [9]. As with traditional Doppler, two or more pulses are sent successively along each scan line of the image and the pairs of echo trains are compared for the presence or absence of a frequency shift (indicating movement). However, unlike traditional Doppler, power Doppler ignores the direction and velocity of the moving structure; if a frequency shift is detected, indicating motion of the structure, then colour is displayed as an overlay whose saturation is related to the amplitude of the echo which has moved. If no frequency shift (movement) is detected, no colour is displayed.

**Figure 3:**
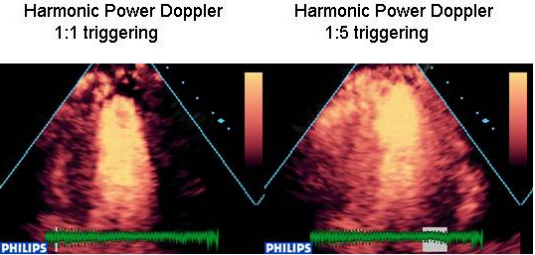
Harmonic power Doppler imaging [see also additional file 1]

This method is ideally suited to high mechanical index destructive imaging, as the first pulse destroys the myocardial micro-bubbles, generating a brief, high amplitude echo rich in harmonics. The second pulse finds that the bubbles have 'moved', and thus colour is overlaid on the echo image over the areas of myocardium that contained micro-bubbles. In an area with no micro bubbles, there is no 'movement' recognised and there is no colour overlay applied to that region. Note that the technique is not actually detecting movement of the bubbles in the circulation, but rather their destruction. This technique has been correlated in human subjects with SPECT[10] and has been used extensively in clinical trials (Figure 3 and additional file 1).

The most important limitation of this technique is motion artefact from tissue, because tissue motion will be expressed like bubble destruction, potentially showing perfusion when none is present (a false negative). If Doppler frequency is increased, pulse separation is reduced, so tissue movement between pulses can be minimized. However, if the pulses are too close, not all the gas within the bubble will have dissipated before arrival of the next pulse, so the difference between pulses is reduced, possibly leading to false positive perfusion defects. Air-filled micro-bubbles are optimal for this technique because of rapid dissipation of the gas, allowing closely spaced pulses.

##### Pulse Inversion Doppler

**Figure 4:**
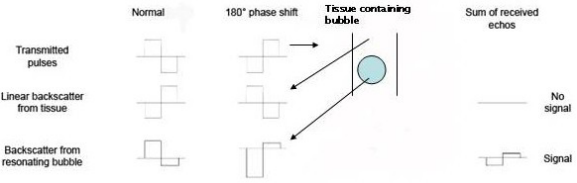
Pulse inversion Doppler.

Another grey scale high MI technique is pulse-inversion imaging whereby two beam mode pulses are sent in rapid succession into the myocardium. The second pulse sent is a mirror image of the first (i.e. 180° phase shift). The scanner processes the consecutive returning pulses by adding them together. Tissue generates a linear echo, thus the addition of one pulse to the other should cancel out to zero and no signal is generated. Micro-bubbles produce non-linear echo signals and the summation of returning pulses will not equal zero and a signal will be registered (Figure 4).

Using this technique, processing can theoretically be limited only to signals generated by bubbles. As well as being a grey-scale technique, tissue motion artefacts are a major limitation, as movement of tissue also creates non-linear signals. Nevertheless the theory behind this technology has led to the development of successful real time imaging.

##### Ultraharmonic imaging

With the development of increased bandwidth transducers, another intermittent high MI technique has evolved to improve the contrast to tissue ratio of backscatter. While intermittent harmonic imaging demonstrates myocardial perfusion, it is limited by the presence of tissue signals at the second harmonic. However, bubble destruction causes backscatter at 3rd 4th and sub-harmonics. At these higher harmonics the tissue signal is negligible, thus processing only backscatter from these further harmonics enables selective enhancement of the contrast signal. Interestingly, current ultra-harmonic imaging involves processing signals from between the second and third harmonic.

The strength of the high MI approaches are their sensitivity for the presence of contrast, because bubble destruction results in the highest amplitude backscatter. The disadvantages are that they lack simultaneous assessment of function, require reliable ECG triggering and image acquisition, and can be both technically challenging and time consuming (of particular importance for stress imaging). While the difficulty maintaining image position with long triggering intervals has been aided by the use of low MI localization images, respiratory movement is almost inevitable.

#### Low Power Techniques – Real Time Imaging

**Figure 11:**
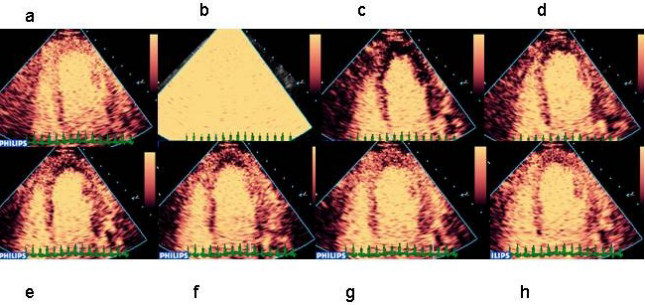
Destruction replenishment imaging with real time MCE – see text.

In recent years, technologies have been developed which so successfully exploit the non-linear responses of micro-bubbles, that even low amplitude micro-bubble backscatter can be isolated from tissue signals for processing. This allows continuous low power imaging to be performed, with limited bubble destruction, enabling simultaneous assessment of wall motion and perfusion in real time (although frame-rate is minimized in order to reduce bubble destruction) (See table 1). Hence low MI imaging is commonly known as real time imaging,

The use of low MI has two major benefits; (1) The bubbles undergo stable non-linear oscillation emitting continuous fundamental and harmonic signals and (2) the tissues themselves do not generate harmonic signals at low MI. Like incremental triggered imaging, low MI imaging enables assessment of micro-bubble replenishment of the myocardium over time after bubble destruction. With low MI imaging, myocardial bubble destruction is achieved by transmission of a series of a high MI pulses ("flashes"), after which replenishment can be observed in real time (see figure 11). There are 2 major real time techniques.

##### Power pulse inversion imaging

This technique combines the non-linear detection performance of pulse inversion with the motion discrimination capability of power Doppler. Multiple transmit pulses of alternating polarity are used, and Doppler signal processing techniques are applied to distinguish between bubble backscatter and backscatter from tissue. In a typical configuration, echos from a train of pulses are combined in such a way that signals from moving tissue are eliminated (see also figure 4).

##### Power modulation imaging

This method utilises the same signal subtraction principles, as PPI, with the transmitted pulses identical in phase but of different in amplitude or 'power' – hence power modulation, one impulse of full power, the other half that power. Echos reflected from stationery tissue are linear, thus if we subtract two times the lower power from full power, the signals should cancel out whereas a non-linear oscillation of micro-bubbles will generate a signal. In power modulation, fundamental imaging is most suited, because the tissue subtraction technique is so effective that the best signal to noise ratio from contrast to tissue is at the fundamental frequency. Both techniques have been validated in animals and utilised for qualitative assessment of perfusion in humans[11,12].

### Bubble administration

The decision regarding the use of a bolus or infusion for intravenous administration of micro-bubbles is dependant on a variety of factors, including the type of micro-bubble used, equipment and staff available and clinical indication for the test (LVO, qualitative perfusion or quantitative perfusion). For MCE, bubbles should be infused with the aim of LV opacification and adequate myocardial perfusion with minimal/no attenuation of the basal segments. The amount of contrast required for this varies from bubble to bubble, machine to machine and technique used (intermittent or continuous), as well as showing patient to patient variability. Ideally all studies, particularly those assessing perfusion would involve a continuous infusion of micro-bubbles, with a mechanically controlled infusion preferable to a manually controlled one (slow continuous injection). This enables establishment of a true steady state for optimal imaging and in particular quantification.

## II. Left ventricular opacification

Accurate evaluation of regional and global left ventricular function by echocardiography is dependent on adequate endocardial border resolution. Using fundamental imaging, approximately 20% of resting echos demonstrate inadequate endocardial definition [13], defined as ≥ 2 segments not seen at baseline. While native tissue harmonic imaging enables better endocardial definition than standard fundamental imaging and reduces the number of patients with inadequate studies to 5–10%, contrast induced LVO still confers benefit over harmonic imaging [14]. The most challenging patients have obesity, chronic lung disease or chest wall deformities. Ventilated patients in intensive care also provide significant difficulties because of patient positioning and compliance.

Techniques that enhance discrimination between myocardial tissue and the blood pool may therefore improve the clinical utility and diagnostic accuracy of echo. Left ventricular opacification (LVO) by contrast echo enhances this discrimination to better define the endocardial surface.

**Figure 5:**
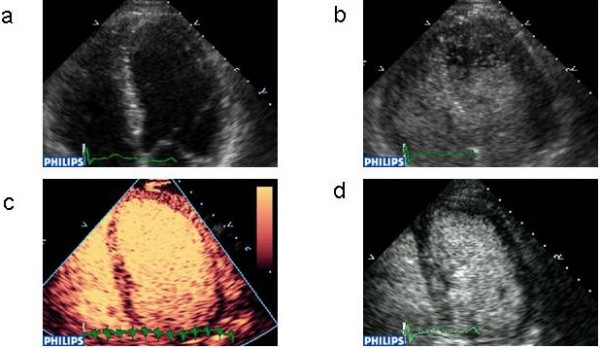
Importance of machine settings for using contrast for LV opacification. (a) In this example, endocardial border definition is probably adequate with standard tissue harmonic imaging – [see additional file 2.] (b) The use of contrast for LVO with standard diagnostic harmonic imaging machine settings provides worse border definition in the lateral wall, and apical bubble destruction, illustrating the importance of appropriate machine settings – [see additional file 3]. Image (c) shows machine settings for myocardial perfusion imaging – this provides assessment of myocardial perfusion and wall motion, but the frame rate for WMA is 20–25 Hz and thus subtle WMA's could be missed – [see additional file 4]. Therefore for optimal assessment of WMA image (d) displays specific intermediate MI imaging at high frame rate designed specifically to enhance the endocardial/cavity border. Even in this example there is some apical swirling despite the focal zone set in the mid LV – [see also additional file 5].

Contemporary LVO involves administration of perflurocarbon micro-bubbles (which show superior duration of opacification and enhancement of endocardial definition compared with air filled bubbles [15]), and intermediate MI harmonic imaging (0.4–0.5) which allows continuous high frame rate imaging, results in reduced bubble destruction, leads to production of micro-bubble harmonic signals with minimal tissue harmonic production, enabling maximal discrimination between the opacified blood pool and myocardium (Figure 5 and additional files 2, 3, 4, and 5).

### LV volume measurement

While there is clear evidence that contrast LVO can improve endocardial border definition, there are theoretical reasons why this may not necessarily translate into more accurate assessment of LV cavity size, volume and function. Attenuation artefacts sometimes obscure the endocardial surface, particularly in the basal segments and myocardial contrast may confuse distinction of the border between the blood pool and the endocardium. Most importantly, the use of any 2-D echo technique to assess left ventricular volumes and ejection fraction (with or without contrast), requires a standard imaging plane. Fore-shortened views or views not oriented through the centre of the ventricular cavity will lead to an underestimation of volume no matter how good the endocardial border definition [16].

**Figure 6:**
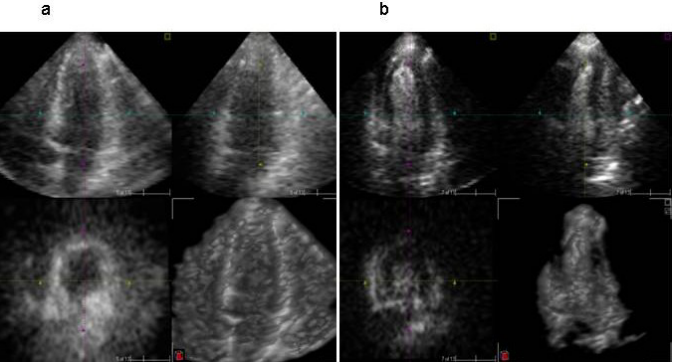
Realtime 3D echocardiography without (a) – [additional file 6], and with contrast enhancement (b) [additional file 7]. There is clear benefit for LV border detection. [See also additional files 8 and 9 for real time 3-D movies without and with contrast respectively.]

Notwithstanding these potential technical limitations, contrast LVO using fundamental imaging improves the correlation between LV volumes obtained with echo and MRI [17]. In addition, accurate classification of systolic function by calculated ejection fraction has been improved from 70 to 94% by the use of contrast, with this improved accuracy almost exclusive to patients with poor baseline images. A subsequent study confirmed that, even using harmonic imaging, the measurement of LV volumes was optimised with contrast LVO, using electron beam CT as the standard of reference [18]. While the role of LVO for 3D imaging is currently undefined, it seems likely that this will be an important application of contrast echo (Figure 6 and additional files 6, 7, 8 and 9).

### LVO and wall motion analysis

Enhanced endocardial border definition can improve the accuracy and inter-observer agreement for assessment of regional wall motion at rest [19]. Compared with MRI, the number of segments visualized was clearly improved after contrast (86% visualised before contrast, 99% after contrast). In addition, identification of segments with abnormal wall motion improved 82% to 100%, with the clinical utility of the contrast being greatest at the lateral and anterior walls. Importantly, the inter-observer agreement for assessment of individual wall segments was significantly improved and contrast also improved intra-observer agreement for determination of normal versus abnormal wall motion and assessment of the severity of wall motion abnormality compared with MRI. The ability of LVO to improve scoring and inter-observer variability of regional wall motion at rest has important implications for stress echocardiography.

Stress echocardiography is an established clinical tool with a high sensitivity and specificity for the diagnosis of coronary artery disease (CAD). During stress echo the diagnosis of CAD is based on detection of regional contractile dysfunction, and requires visualization of all myocardial segments to document or exclude abnormalities definitively. Reduced endocardial border definition is exacerbated during stress because of chest wall movement during hyperventilation and cardiac translational movement during tachycardia. With fundamental imaging, inadequate endocardial definition has been reported in up to 30% of stress echos [20]. In addition, Hoffman et al demonstrated that suboptimal studies have worse reproducibility and a poorer inter observer variability, with inter-institutional institutional observer agreement as low as 43% for studies with poor image quality[21] Tissue harmonic imaging, digital side by side analysis and standardised reporting criteria have alleviated but not overcome this problem[22].

Wall motion scoring and reproducibility during stress echo were even improved with air filled contrast agents and fundamental imaging[23]. Perflurocarbon filled agents demonstrated almost complete and consistent endocardial border definition [24], with superiority even to tissue harmonic imaging, and the greatest improvement being seen in patients with poorest image quality (26).

**Figure 7:**
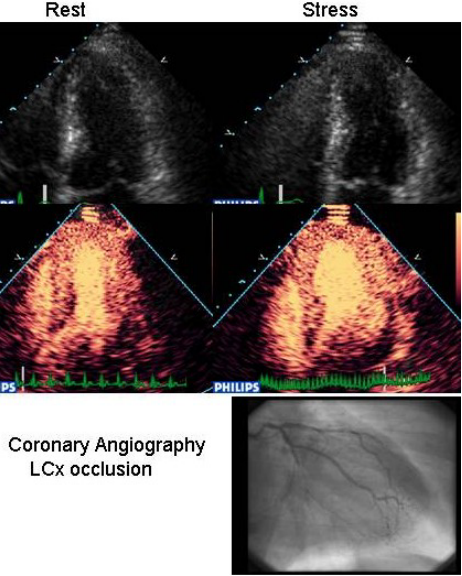
End systolic frames of 4CV at rest (left) and post stress (right). Note there is no obvious difference in the shape of the cavity on the grey scale images. Importantly, the LVO images demonstrate a clear change in shape with the basal and mid lateral segments lagging, suggestive of LCx stenosis. In addition, the mid lateral segment has a perfusion defect which was not present at rest. Subtotal occlusion of the LCx was demonstrated at angiography. [See also additional files 10-23 for entire study].

Despite these clear advantages, the critical clinical question of whether LVO actually improves the accuracy of stress echo for diagnosis of CAD remains unanswered – Dolan [25] came closest by investigating 229 patients undergoing dobutamine echo followed by angiography, the largest study of contrast stress echo (for LVO) with angiography as the gold standard. As previously documented, the endocardial border definition and inter observer variability was superior with contrast. Their important finding was that there was comparable sensitivity, specificity and accuracy for the presence of coronary disease between patients with poor resting images using contrast LVO and those in whom standard imaging gave good resting images. Moreover, our recent work shows that the improvement of LVO is an important contributor to the incremental benefit of MCE (Figure 7 and additional files 10,11,12,13,14,15,16,17,18,19,20,21,22, and 23).

Thus, left ventricular opacification by contrast echo:

(1) improves the visualization of myocardial endocardial border definition;

(2) improves the accuracy of ventricular volume assessment and estimation of ejection fraction compared with standard fundamental and harmonic imaging.

(3) improves the ability to identify and grade resting wall motion abnormalities

(4) provides superior endocardial visualization while imaging at peak dobutamine stress and can enable the accuracy of a dobutamine stress echo in a technically difficult patient to be at least as good as that of a patient with good resting images;

(5) reduces the inter-observer variability for all of the above.

From an economic standpoint, the use of contrast agents during stress echo has been calculated to be cost effective [26] with the cost of the contrast agent itself more than offset by savings incurred by reducing downstream repetitive testing, improved laboratory efficiency and a lower rate of false positive and negatives. However, the calculations are based on a formulas incorporating improved accuracy of the technique for the diagnosis of CAD, compared with standard imaging, which remains un-proven.

**Figure 8:**
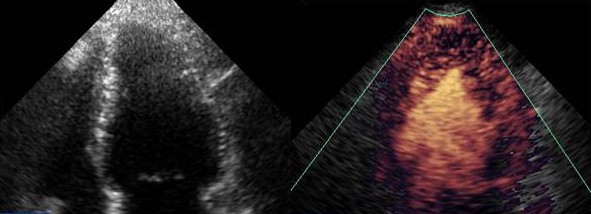
Use of contrast echo to identify a patient with apical HCM. Note the 'spade shaped LV cavity' on the contrast image [See also additional files 24 and 25]

In summary, the use of contrast for LVO is justified for standard or stress imaging of technically difficult patients, and possibly, for calculation of ventricular dimensions in patients whom accurate quantitative serial follow up is critical, eg. chemotherapy or valvular heart disease. Other clinical uses of contrast for left ventricular opacification include confirming or excluding the presence of left ventricular thrombus and delineating other left ventricular structures like pseudoaneurysm, apical hypertrophic cardiomyopathy (Figure 8 and additional files 24,25), and non-compacted left ventricles [27-29].

## III. Myocardial contrast echocardiography

### The ischaemic cascade

In the ischaemic cascade, hypoperfusion precedes wall motion abnormalities, which precede ECG changes and the onset of chest discomfort [30]. Stress echo, which provides an indirect marker of hypo-perfusion by recognition of wall motion abnormalities, is more than 80% sensitive and specific for detection of CAD [20]. The technique has two groups of limitations;

1) Interpretive. Wall motion analysis is fundamentally subjective [31], leading to the need for specific training and discordance between observers. There are particular problems in the assessment of the inferoposterior wall.

2) Dependence on the induction of ischaemia (increased myocardial oxygen demand) to provide diagnostic information. Many problems spring from this – dependence on performance of adequate stress, influence by beta blockade, limited sensitivity for mild (eg single vessel) CAD, poor capacity to identify the extent of CAD (eg. 50% sensitivity for the detection of multi vessel disease)[32].

SPECT perfusion imaging is the best-established clinical method to address perfusion directly and theoretically should be more sensitive than stress echo because hypoperfusion occurs earliest the ischaemic cascade. However, the difference between the methods is marginal because of the technical limitations of SPECT scanning, which also relies on subjective interpretation.

If technically feasible there is clear role for echocardiography to assess myocardial perfusion. Ultrasound is more widely available, portable, avoids radiation and has a better spatial resolution than SPECT scanning (up to 1 mm, compared with >10 mm). An ideal technique would allow assessment of myocardial perfusion and wall motion simultaneously.

### Coronary circulatory physiology

Understanding the pathophysiology of normal myocardial perfusion is essential for understanding detection of CAD with MCE. Contrast micro-bubbles act as pure intravascular tracers, traversing the myocardial vasculature, and contrasting with MRI tracers (which escape into the extravascular space) and radionuclides (which enter myocytes).

#### Myocardial blood volume

The coronary blood volume (CBV) encompasses the entire coronary system, which includes the epicardial arteries, the arterioles, the capillary network, venules, veins and the coronary sinus. Approximately one-third of the CBV resides within the ventricular myocardium. This myocardial blood volume (MBV) includes "microvessels" of < 300 microns in diameter, with approximately 90% of the total myocardial blood volume lying within the capillaries (6–7 microns in diameter)[33]. Assessment of myocardial perfusion with MCE involves processing backscattered signals received from micro-bubbles within the MBV, so that myocardial opacification almost entirely reflects backscatter from micro-bubbles flowing through the myocardial capillary compartment.

There are 2 components of this **myocardial blood flow **appreciated with MCE i) the intensity of the backscatter signal from the micro-bubbles within the myocardium (brightness of its appearance on echo) – related to the myocardial blood volume, ii) the rate of increase in intensity after bubble destruction reflects the red blood cell velocity. Their product, represents myocardial blood flow. The response of MBF myocardial blood flow and it's components to stress is central to the application of perfusion imaging.

##### Normal physiology of perfusion

Because of the near complete extraction of O2 from red cells by the myocardium under basal conditions, any increase in myocardial O2 demand must rapidly translate into increases in coronary blood flow. In the absence of a coronary stenosis, when myocardial O2 demand is increased, there is sufficient vasodilator reserve to allow coronary flow to increase by a factor of 4–6 fold above resting levels.

The work of Kaul, Wei and Lindner at the University of Virginia has shown that the ability to increase coronary flow in response to increased demand is achieved through a process of coronary autoregulation, controlled predominantly by the arterioles (See table 2). The coronary micro-circulation strives to maintain a minimum trans-capillary pressure of approximately 30 mmHg. In the absence of a coronary stenosis, a resting patient with a mean aortic pressure of 90 mmHg will have a pre-capillary pressure of ~45 mmHg. This natural resistance between the aorta and the capillaries (of ~45 mmHg) is provided by the arterioles[34] which present up to 60% of the total coronary vascular resistance. In the presence of increased myocardial oxygen demand, there is arteriolar vasodilation, reducing the resistance at the arteriolar level, which enables a higher precapillary pressure, translating into **increased red blood cell velocity **across the capillary network, and opening dormant capillary networks in order to maintain mean trans-capillary pressure, thus **increasing the overall myocardial blood volume**. Hence overall **myocardial blood flow is increased**. Importantly, pure vasodilator stress tends to increase red cell velocity without marked changes in overall myocardial blood volume.

In the presence of a significant, non-critical (60–90%) epicardial artery stenosis at rest, completely **normal **resting blood flow is maintained by arteriolar vasodilation. As a result myocardial perfusion imaging techniques cannot detect defects at rest in patients with non-critical stenoses.

Thus identification of perfusion defects in the setting of a non-critical coronary stenosis requires stress imaging to induce either ischaemia (increased myocardial oxygen demand) or hyperaemia (pharmacologically induced maximal arteriolar vasodilation). During stress we expect a 4–6 fold increase in flow to areas supplied by non-stenotic arteries, mediated by arteriolar vasodilation, increased red cell velocity and increased myocardial blood volume (opening of dormant capillary networks). Flow in the perfusion bed subtended by a significantly stenosed artery is not augmented, as resting arteriolar vasodilation is present, so limited augmentation of flow can be achieved with hyperaemia. In some cases the pre-capillary pressure drops significantly due to low distal coronary pressure and steal. With reduced pre-capillary pressure the only means of attempting to maintain normal trans-capillary pressure is to shut down capillary networks which were previously open (capillary de-recruitment). The net result is that direct comparison between regions subtended by a significant stenosis and normal territories reveals perfusion mismatch.

In the presence of a critical stenosis, maximal arterial vasodilation maintains perfusion at rest. Any further reduction in distal coronary pressure directly translates into reduced precapillary pressure and reduced myocardial blood flow. Patients with critical lesions commonly have collateral vessels, making assessment of resting perfusion a complex phenomenon.

### Qualitative assessment of myocardial contrast for diagnosis of CAD

There are three aspects to myocardial contrast opacification – signal intensity (equivalent to myocardial blood volume), pattern of filling and rate of filling.

#### Signal intensity

Myocardial blood volume is the easiest parameter to interpret qualitatively, and most studies have addressed differences in MBV between rest and stress in myocardial segments. Perfusion has traditionally been scored using a graded scale; 1 for homogenous perfusion, 0.5 for reduced perfusion and 0 for absent perfusion [8]. The presence of a stress induced perfusion defect not seen on the resting images indicates ischaemia, but in the absence of destruction-replenishment imaging, may appear normal in mild disease. The presence of a resting perfusion defect signifies infarction or artefact, with an infarction likely if there are associated regional wall motion abnormalities at rest, and if the defect conforms to the distribution of a coronary vascular territory.

**Figure 9:**
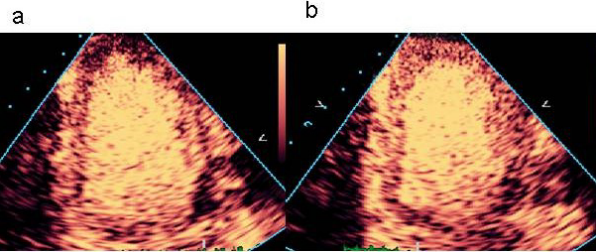
False positive defects with real-time MCE. Pseudo-apical defects are due to apical bubble destruction (a). Relocation of the focus from the base toward the apex (b) leads to "resolution" of the apical abnormality, but use of a mid-ventricular focus placement may lead to more problems with definition of the basal segments. This case also exemplifies attenuation of the basal lateral segment by contrast within the LV cavity.

False positive defects often reflect technical limitations (Figure 9). Failure to obtain signal from a segment may be due to attenuation by overlying contrast, shadowing by rib or other structure, bubble destruction (especially in the apex) or failure to deliver sufficient contrast to enter the microcirculation.

#### Perfusion distribution

**Figure 10:**
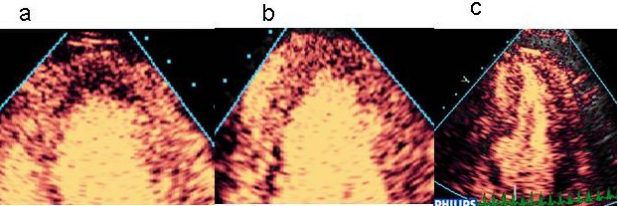
Contribution of regional shape changes to the identification of perfusion defects, including irregular wall contour in the apex (a) and mid-inferior segment (b), both associated with subendocardial defects. The C shape of the basal inferior segment complements the diagnosis of a perfusion defect in this segment. (c)

While most infarctions are shown as a transmural perfusion defect, stress-induced defects are often restricted to the subendocardium (Figure 10a,10b). The detection of this finding allows a greater level of confidence than does a transmural defect, or particularly epicardial defects (which may be artefactual).

The location of a defect has an important relationship to its likelihood of being a true or a false positive. Generally, defects on the left hand side of the image are most likely to be true positives and those restricted to the right side (anterior, lateral wall) should be considered very critically before they are identified as abnormal, as these are the most common sites of false positives. Fortunately, in our experience the apex is a more sensitive marker of LAD disease than the anterior wall, and is usually well seen. Likewise, a lateral segment should be matched with a posterior wall finding before circumflex disease is reported. The basal segments are also problematic – especially with low MI imaging – and in our experience, the basal inferior wall should be matched to an adjacent abnormal segment (basal septum, mid-inferior) to reduce false positive interpretations.

The shape of the LV cavity is a clue to true positive findings. A "C-shaped" perfusion defect in the inferior wall (Figure 10c) is a common distribution with right coronary disease. Irregularities of the walls (Figure 10b) or apex (Figure 10a) or apical "beaking" (Figure 10c) may provide evidence of dyssynchrony, and are often associated with subendocardial perfusion abnormalities.

#### Rate of filling

The rate of replenishment after bubble destruction is dependent on coronary flow, and even if this is not quantified, rate of refill is a reliable and sensitive marker of a true positive defect. Using low MI techniques, micro-bubbles are infused until adequate LVO and myocardial opacification are achieved (Figure 11b). Several pulses of high mechanical index are delivered (Figure 11c), causing destruction of micro-bubbles in the beam elevation, such that the previously opacified myocardium is now empty (Figure 11d). The replenishment time of micro bubbles into the myocardium can be observed qualitatively (Figure 11e,11f,11g,11h).

**Figure 12:**
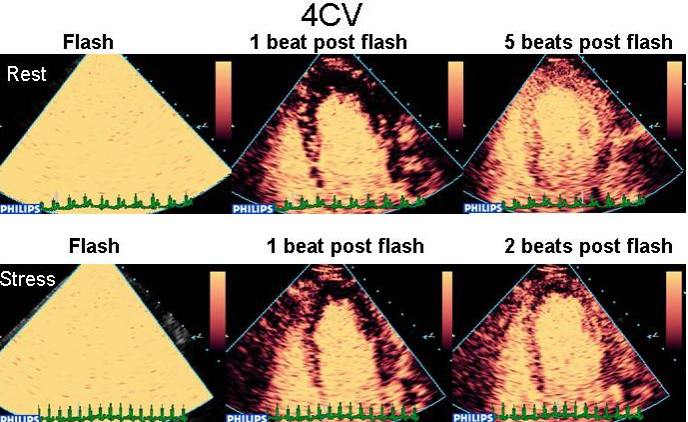
A clear apical defect is evident 2 beats post flash at peak stress (bottom line) which was not evident at rest (top line), consistent with LAD stenosis. [See additional files 26-39 for full case movies, additional file 40 for angiogram and additional file 49 for curve fits].

**Figure 13:**
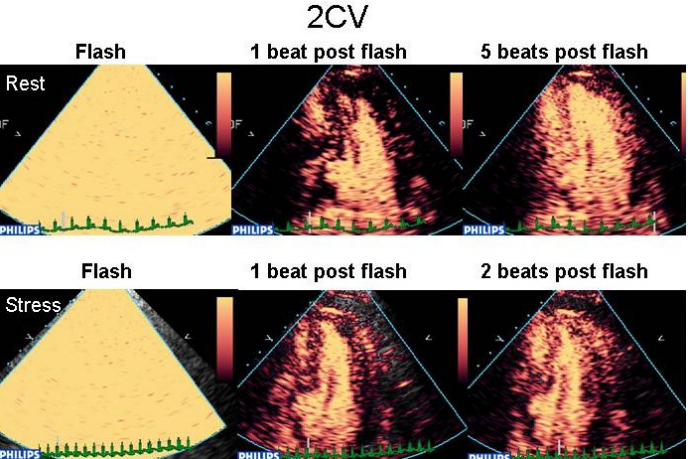
A basal and mid inferior defect is evident 2 beats post flash at peak stress which was not evident at rest, consistent with RCA stenosis. [See additional files 41-46 for movies and 47 and 48 for angiography].

**Figure 14:**
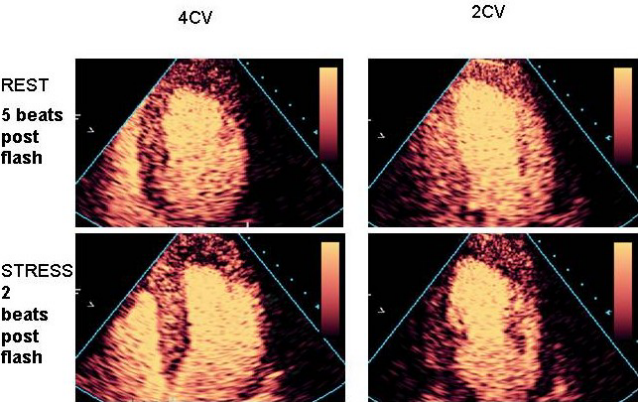
On the 4CV, an apical and a subendocardial basal infero-septal defect are evident 2 beats post stress. The lateral wall is affected by artefact. On the 2CV, there is hypoperfusion of the inferior wall and subendocardial apical defect on the post stress images. This is consistent with multi-vessel disease.

As the mean myocardial microbubble velocity is 1 mm/sec, and the beam elevation is approximately 5 mm, it would take up to 5 seconds (ie 5–6 heart beats at a resting heart rate from 60–80) for homogenous opacification of the myocardium at rest. With hyperaemia in the absence of stenosis, myocardial blood flow should increase 4–6 fold. Thus, images taken within 1–2 seconds following bubble destruction (ie 2–3) heart beats at a peak heart rate of 140) should demonstrate complete homogenous refill within this time frame, and failure to fill in a perfusion defect thus represents reduced myocardial blood flow. Perfusion defects noted on the post stress images, not evident on the resting images suggest ischaemia [35]. -see figures 12, 13 and 14 (and additional files 26,27,28,29,30,31,32,33,34,35,36,37,38,39,40,41,42,43,44,45,46,47,48, and 49) for evidence of LAD, RCA and multi-vessel CAD respectively. Assessment of end-systolic frames is preferable, despite the presence of more coronary flow in diastole, because the myocardium is thicker and there is less risk of contamination by the blood pool, and perhaps also because the capillary network is 'sealed' during systolic contraction with minimal flow in or out of the capillary compartment [36].

High MI techniques apply the same principles, with infusion until homogenous myocardial opacification. Imaging is then changed to a sequential intermittent mode, beginning with continuous high MI imaging (which results in bubble destruction and empties the myocardium). Images are then recorded intermittently, gated from the ECG (usually end systolic) with an incrementally lengthening triggering interval, ie 1:1 an image every cardiac cycle, then 1:2, and image every two cardiac cycles, then 1:3, 1:4 etc. The same principles are used for assessment of perfusion defects. A perfusion defect evident on 1:2 or 1:3 triggering at peak stress not seen at 1:5/6 triggering at rest is significant and reflects an ischaemic response to stress.

### Validation of qualitative contrast echo for diagnosis of CAD

Qualitative assessment of perfusion with MCE was validated in open chest dogs using graded coronary stenosis [37]. The original landmark paper in humans [8] compared MCE (intermittent harmonic MCE with offline digital subtraction and colour coding) with sestamibi SPECT scanning in 30 patients undergoing dipyridamole stress to investigate known or suspected coronary disease. Normal segments showed a 91% concordance, and abnormal segments an 85% concordance, with 90% concordance by vascular territory. There was an overall 86% concordance for the detection of coronary disease. Subsequent studies have validated MCE following vasodilator or dobutamine stress using other imaging modalities, including power Doppler and real time power pulse inversion/power modulation in animals and in humans have produced similar results to SPECT [38-40]. A recent study using real time imaging was the first to report the use of MCE with exercise stress [41] (which is technically challenging because of cardiorespiratory movement and short duration of hyperaemia) and again demonstrated concordance between MCE and SPECT imaging. There was a 76% agreement between MCE and SPECT and an 88% agreement between the combination of wall motion and MCE with SPECT. This study incorporated the wall motion data from real time imaging, and despite the low frame rate, the results hint at the incremental benefit of combining the approaches.

While numerous studies examining various stress and imaging modalities have demonstrated concordance between MCE and SPECT, there remains a paucity of data using quantitative coronary angiography (QCA) as the gold standard. The presence and extent of CAD by QCA has traditionally been used as the reference standard for the assessment of CAD, but few studies have compared MCE and angiography (Table 3) [42]. In 45 patients undergoing mainly dobutamine stress echocardiography, Cwajg et al showed real time contrast-enhanced imaging was more sensitive than standard stress echocardiography (87% vs 56%), and gave a better recognition of disease extent (85% vs 39%). These results were surprisingly unfavourable for standard imaging, perhaps reflecting the limitations of lower frame-rate for real-time imaging. Moreover, no specificity data were available from this study. In the study of exercise stress (41), only 44 of the 100 patients who had MCE and SPECT proceeded to angiography, and in this group, the sensitivity and specificity of wall motion and MCE were not significantly different. In a recent study of 85 prospectively recruited patients, the largest involving quantitative coronary angiography, we demonstrated the addition of contrast (for LVO and MCE), to standard ExE significantly enhanced the sensitivity of the test for detection of CAD from 74 to 91%, with a non-significant reduction in specificity (57).

**Table 3:** Myocardial contrast echo studies with patients undergoing coronary angiography.

Author	CAD	No CAD	Sensitivity MCE	Specificity MCE	Frequency of angiography
Cwajg [42]	32	13	87%	_	All 45
Shimoni [41]	28	16	75%	100%	44 (44%) of 101
Heinle [10]	12	3	75%	67%	15 (12%) of 123
Wei [43]	15	-	100%	-	15 (28%) of 54
Rocchi [55]	12	-	89%	-	12 (48%) 25
Olszowska [56]	44	-	97%	-	All 44
Moir [57]	43	27	91%	70%	70 of 85

### Quantitative myocardial contrast echo for diagnosis of CAD

Like stress echo and nuclear perfusion imaging, MCE is also limited by the qualitative nature of the interpretation. Subtle differences in video intensity between vascular beds may not be visually evident, potentially reducing the sensitivity for the detection of stenosis. Quantitative methods may help to alleviate this limitation and possibly reduce intra-observer and inter-observer variability in assessment.

**Figure 15:**
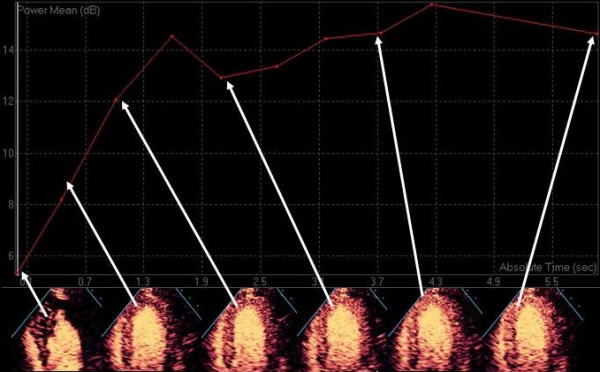
The raw signal intensity data from the apical septal segment is plotted against time after destruction.

**Figure 16:**
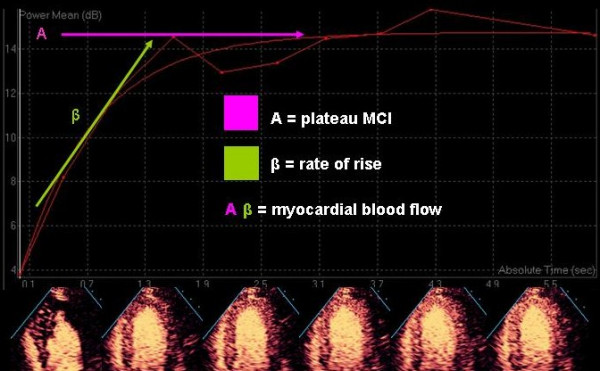
an exponential function curve is applied to allow calculation of A, beta and A*beta.

The process of quantitative myocardial contrast echo was validated in open chest dogs using intermittent imaging, and the same destruction-replenishment approach is used for contemporary qualitative assessment [43]. At a steady state during continuous intravenous infusion of micro bubbles, the number of bubbles entering or leaving any capillary unit is constant and depends on the flow rate of the bubbles. If the micro bubbles are destroyed at time zero, the video intensity in a selected myocardial region is close to zero dB (black). With time, bubbles will replenish the beam elevation – the degree of replenishment into the beam elevation increases as the time after destruction is increased until eventually the entire ultrasound beam elevation is replenished and a plateau is reached, whereby no further increase in video-intensity/brightness can occur (Figures 15 and 16). Because this relationship was originally described with intermittent imaging, it is usually described in terms of video-intensity vs pulsing intervals.

The video intensity vs pulsing interval or time curve resembles an exponential growth function that can be fitted by the equation I = A (1-e-βt) where y is the video intensity at the pulsing interval or time t, A is the plateau video intensity and β represents the rate of rise of the slope. In this model the ***plateau myocardial video intensity ***(A) represents the **myocardial blood volume**. The rate of rise of the slope (β) represents the mean myocardial **red blood cell velocity **and their product (A*β) represents **myocardial blood flow**. Wei demonstrated an excellent linear relationship between absolute myocardial blood flow measured with radiolabelled microspheres and myocardial contrast echo derived myocardial blood flow. While ECG gated intermittent triggered harmonic imaging was used to validate these measurements, both power Doppler and real time techniques have subsequently demonstrated similar results in animal experiments ([44-46]).

More recently, Wei evaluated the ability of MCE to calculate flow reserves from these measurements in humans ([47]), using intermittent imaging in 30 selected patients undergoing coronary angiography (11 of whom had no CAD and 19 had non-critical single vessel stenoses). Quantitative MCE and invasive measurement coronary flow (Doppler flow wire) were performed at rest and following vasodilator stress. In the normal subjects, myocardial blood flow velocity (β) and myocardial blood flow (A*β) reserves demonstrated a linear relationship to coronary blood flow reserve measured invasively. In patients with CAD, there were significant differences in MBF velocity reserves between patients with mild moderate and severe stenoses, and a MBF velocity reserve of < 1.8 indicated a >70% stenosis. Thus MBF reserve appears to be a feasible non-invasive measure of CBF reserve in humans, which may allow non-invasive assessment of CAD and micro-vascular dysfunction. More recently, Dawson investigated the use of quantitatively derived MBF reserves to diagnose CAD using SPECT as the gold standard. She demonstrated moderate feasibility, with quantitative MBF reserves from both high and low MI imaging able to identify perfusion defects ([48]). Low MI imaging had a lower sensitivity.

### Contrast Echo and myocardial viability in chronic CAD

It is now well recognised that regional or global ventricular dysfunction does not necessarily imply irreversible necrosis. Hypokinetic, akinetic or dyskinetic, yet viable myocardium may be stunned or hibernating. Myocardial stunning occurs after a period of acute ischaemia, despite restoration of completely normal blood flow. The natural history of stunning is of spontaneous improvement in the viable myocardium over time. Hibernating myocardium is the term used to describe the presence of significant ventricular dysfunction in patients with chronic CAD, which recovers after revascularisation. Improvement in function of sufficient numbers of viable but hibernating segments is associated with symptomatic benefit and improved survival[49]. Sadly, many patients with chronic CAD and LV dysfunction have minimal viability, and revascularisation of these patients is associated with significant risk, minimal benefit and possibly worse outcome, hence the need for a reliable test for identification of viability.

**Figure 17:**
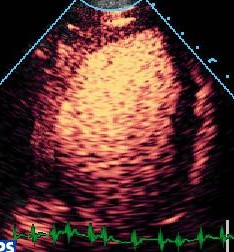
Resting apical 2-chamber view, 10 beats post-flash, demonstrating absent perfusion to the anterior myocardial wall.

Radionuclide scanning, dobutamine echocardiography, MRI and PET scanning are currently available modalities, each with various advantages and disadvantages but similar efficacy for prediction of myocardial functional recovery after revascularization. MCE may also have an important role in this clinical setting. Viable myocardium is associated with preservation of the micro-vasculature, and as micro-bubbles act as pure intra-vascular tracers, the presence of myocardial perfusion by any MCE technique at rest implies viability (see figure 17). Using intra-coronary injection of bubbles, Nagueh demonstrated MCE was feasible and had similar accuracy to thallium SPECT and dobutamine echo for identification of functional recovery[50]. Using intravenous micro-bubble administration, MCE demonstrated comparable efficacy to SPECT and DSE. Importantly in both of these studies quantitative MCE was superior to qualitative assessment[51]. Unfortunately there remains a paucity of further data in this clinically important area.

### Safety of contrast echocardiography

The incidence of reported adverse events in human trials (mainly investigating LV opacification) has been very low. There have been particular theoretical concerns raised about histologic abnormalities and cardiac marker elevation in animals with high MI imaging[52], and whilst serum tropinin levels are normal in humans after high MI imaging[53], recent work has demonstrated troponin I and myoglobin in coronary sinus samples of humans after high MI imaging[54].

In recent months the agent SonoVue (a phospho-lipid shell encapsulating sulphur hexafluoride gas), approved in Europe, was withdrawn from clinical use because of adverse events including fatalities related to idiosyncratic hypersensitivity reactions. At this stage, specific details about the dose of agent and imaging modality used in these cases is not available.

## IV. Conclusion

Advances in micro-bubble development, combined with the development of contrast specific imaging modalities have enabled not only excellent LVO, but reliable qualitative and quantitative assessment of myocardial perfusion by ultrasound, following intravenous injections of micro-bubbles. Use of this technology during stress echo increases sensitivity and improves the non-invasive evaluation of CAD.

**Table 1:** Technical details and clinical implications of contrast-specific imaging techniques

**High Power Intermittent Imaging**	Acquisition difficulty	Amount of contrast used	Amplitude of micro-bubble backscatter	Dynamic range	Greyscale or colour	Processed Backscatter	Myocardial perfusion	Wall motion	Artefacts
Tissue Harmonic Imaging	challenging	low	high	wide	grey	harmonic	yes	no	yes
Ultraharmonic	challenging	low	high	wide	grey	harmonic	yes	no	yes
Pulse inversion	challenging	low	high	wide	grey	harmonic	yes	no	yes++
Harmonic Power Doppler	challenging	low	high	wide	colour	harmonic	yes	no	yes
**Low Power Continuous Imaging**									
Power Pulse inversion	**easy**	**high**	**low**	**narrow**	**colour**	harmonic	**yes**	**yes**	**yes**
Power modulation	**easy**	**high**	**low**	**narrow**	**either**	**fundamental**	**yes**	**yes**	**yes**

**Table 2:** Relationship between stenosis severity and stress on capillary pressure, myocardial blood volume and red cell velocity. Arbitrary numbers given for illustrative purposes only.

	**Distal coronary artery pressure**	**Arteriolar resistance**	**Pre-capillary pressure**	**Trans-capillary pressure**	**RBC velocity**	**MBV**
No stenosis REST	90 mm Hg	Normal 45 mmHg	45 mmHg	30 mmHg	Normal	Normal
No stenosis STRESS	90 mm Hg	↓ Maximal 25 mmHg	65 mmHg	30 mmHg	↑	↑
Stenosis REST	75 mm Hg	↓ Sub-maximal 30 mmHg	45 mmHg	30 mmHg	**Normal**	**Normal**
Stenosis STRESS	60 mm Hg	↓ Maximal 25 mmHg	35 mmHg	30 mmHg	↓	↓

## Supplementary Material

Additional File 1 Power Doppler 4CV at rest in normal subject. Click here for file

Additional File 2 A4CV standard harmonic imaging Click here for file

Additional File 3 Contrast LVO with standard harmonic imaging Click here for file

Additional File 4 Real time MCE with LVO and myocardial perfusion. Click here for file

Additional File 5 Contrast LVO with ideal machine settings to optimize image quality and frame rate Click here for file

Additional File 6 3-D non contrast imaging Click here for file

Additional File 7 3-D imaging with contrast Click here for file

Additional File 8 Real time 3-D without contrast Click here for file

Additional File 9 Real time 3-D with contrast. Click here for file

Additional File 10 Mr CC PLAX view rest Click here for file

Additional File 11 Mr CC PLAX view stress Click here for file

Additional File 12 Mr CC PSSAX view rest Click here for file

Additional File 13 Mr CC PSSAX view stress Click here for file

Additional File 14 Mr CC A4CV rest Click here for file

Additional File 15 Mr CC A4CV stress Click here for file

Additional File 16 Mr CC A2CV rest Click here for file

Additional File 17 Mr CC A2CV stress Click here for file

Additional File 18 Mr CC ALAX view rest Click here for file

Additional File 19 Mr CC ALAX view stress Click here for file

Additional File 20 Mr CC contrast 4CV rest Click here for file

Additional File 21 Mr CC contrast 4CV stress Click here for file

Additional File 22 Mr CC contrast 2CV rest Click here for file

Additional File 23 Mr CC contrast 2CV stress Click here for file

Additional File 24 Apical HCM standard 4CV Click here for file

Additional File 25 Apical HCM contrast rest Click here for file

Additional File 27 Mr JD Rest PSAX Click here for file

Additional File 28 Mr JD Rest A4CV Click here for file

Additional File 26 Mr JD Rest PLAX Click here for file

Additional File 29 Mr JD Rest A2CV Click here for file

Additional File 30 Mr JD Rest ALAX view Click here for file

Additional File 31 Mr JD Rest contrast 4CV Click here for file

Additional File 32 Mr JD Rest contrast A2CV Click here for file

Additional File 33 Mr JD Stress PLAX Click here for file

Additional File 34 Mr JD Stress PSSAX Click here for file

Additional File 35 Mr JD Stress A4CV Click here for file

Additional File 36 Mr JD Stress A2CV Click here for file

Additional File 37 Mr JD Stress ALAX view Click here for file

Additional File 38 Mr JD Stress contrast 4CV Click here for file

Additional File 39 Mr JD Stress contrast 2CV Click here for file

Additional File 40 Mr JD angiogram. Click here for file

Additional File 41 Mr T 4CV rest Click here for file

Additional File 42 Mr T 4CV stress Click here for file

Additional File 43 Mr T 2CV rest Click here for file

Additional File 44 Mr T 2CV stress Click here for file

Additional File 45 Mr T 2CV contrast rest Click here for file

Additional File 46 Mr T 2CV contrast stress Click here for file

Additional File 47 Mr T cath LCA Click here for file

Additional File 48 Mr T cath RCA Click here for file

Additional File 49 Mr JD curve fits A4CV Click here for file
