# Supplementary figures and images for: Combination of contrast with stress echocardiography: A practical guide to methods and interpretation
Source: Cardiovasc Ultrasound. 2004 Aug 26;2:15. doi: 10.1186/1476-7120-2-15 (PMC516786; doi:10.1186/1476-7120-2-15)

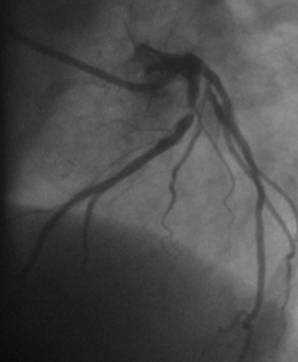

Supplement: Additional File 40 — Mr JD angiogram. [file 1476-7120-2-15-S40.jpeg]

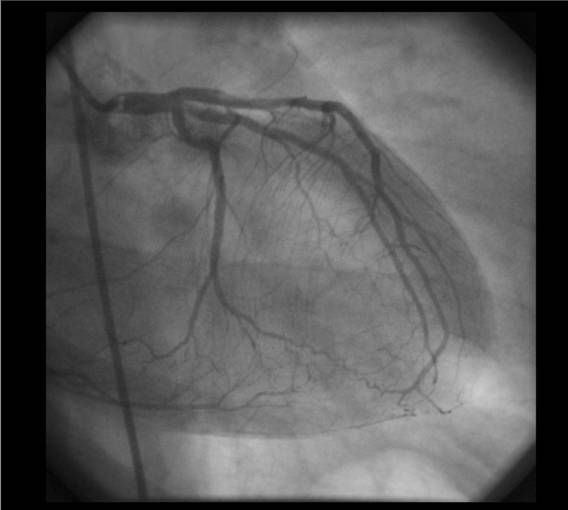

Supplement: Additional File 47 — Mr T cath LCA [file 1476-7120-2-15-S47.jpeg]

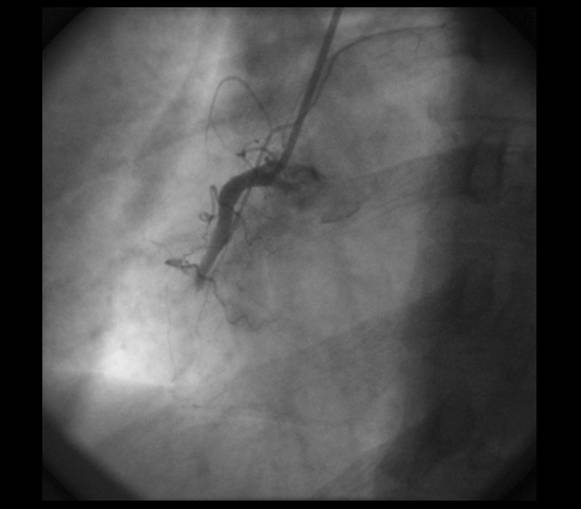

Supplement: Additional File 48 — Mr T cath RCA [file 1476-7120-2-15-S48.jpeg]

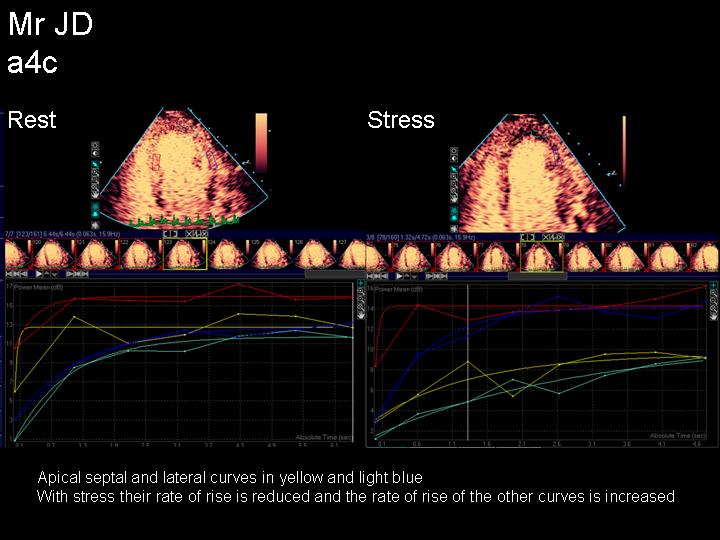

Supplement: Additional File 49 — Mr JD curve fits A4CV [file 1476-7120-2-15-S49.jpeg]
